# Influence of *In-Situ* Oil Sands Development on Caribou (*Rangifer tarandus*) Movement

**DOI:** 10.1371/journal.pone.0136933

**Published:** 2015-09-08

**Authors:** Tyler Muhly, Robert Serrouya, Eric Neilson, Haitao Li, Stan Boutin

**Affiliations:** 1 Alberta Biodiversity Monitoring Institute, Department of Biological Sciences, University of Alberta, Edmonton, Alberta, Canada; 2 Stantec, Environmental Services, Sidney, British Columbia, Canada; 3 Department of Biological Sciences, University of Alberta, Edmonton, Alberta, Canada; The Ohio State University, UNITED STATES

## Abstract

In-situ oil sands development (ISD) involves a network of facilities, wells, roads and pipelines to extract and transport subsurface bitumen. This technology is rapidly expanding and there is uncertainty whether ISDs restrict animal movement, leading to increased extinction probabilities for some wide-ranging species. Here we test for effects of simulated future (i.e., 50 years from now) and current ISDs on simulated movements of woodland caribou (*Rangifer tarandus*), a threatened species across North America. In simulations of future scenarios, we varied the spacing and permeability of ISDs and the presence/absence of protected areas. Permeability was measured as the number of times simulated caribou crossed ISDs with different levels of modelled permeability. We estimated the effects of these factors on caribou step length and annual home range size, key metrics of small and large spatiotemporal scales of movement, respectively. Current caribou crossings of above-ground pipeline features of ISDs were measured using camera traps and compared to expected caribou crossing rates based on present-day caribou movement simulations. Current crossing rates were evaluated within the context of predicted future crossing success rates necessary to maintain caribou step lengths and home ranges. With few exceptions, permeability across ISDs was the main factor affecting caribou movement, more so than spacing between developments or the presence of protected areas. However, minimal permeability (crossing rates of c. 15% to 60%, relative to an undisturbed site was needed to maintain existing home range size and step lengths. The effect of permeability on home range size and step length was non-linear, suggesting that small increases in permeability would provide a disproportionately greater benefit to caribou movement. Our predictions demonstrate that maintaining permeability across ISDs is more important than spacing between leases or including protected areas, and thus provides clear direction for mitigation efforts for features that will exist on the landscape for decades to come.

## Introduction

The desire to have a plentiful source of domestic oil has prompted governments to facilitate the extraction of non-conventional oil reserves [1]. Some of these emerging technologies have the potential to substantially alter the configuration of terrestrial ecosystems. “*In-situ*” methods of oil extraction are increasingly used to access subsurface bitumen, with associated infrastructure consisting of wells, roads, processing facilities, and especially a network of above-ground pipelines used to extract and transport bitumen. Boreal caribou, (*Rangifer tarandus caribou*), a threatened ecotype of woodland caribou, is in rapid decline in Alberta, Canada [2, 3], and has considerable overlap with bitumen deposits where development is expected to intensify [4]. The proximate reason for the decline of woodland caribou is predation through apparent competition with other ungulates [5–7]. However, there is concern that above-ground pipelines will exacerbate caribou declines by restricting their movements [8]. Movement is critical for many wide-ranging species, including caribou, to access resource patches for their survival [9–11]. Restricted movement and thus restricted access to resource patches has been shown to increase extinction probability and decrease lifetime reproductive success for some species that use broad areas for their life-history requirements [12, 13]. *In-situ* developments (ISDs) may limit caribou access to resources, particularly predator-free space, which could ultimately have negative implications for caribou populations. Reduced movements have also led to fragmented populations with subsequent genetic drift for many caribou subpopulations [14].

**Fig 1:**
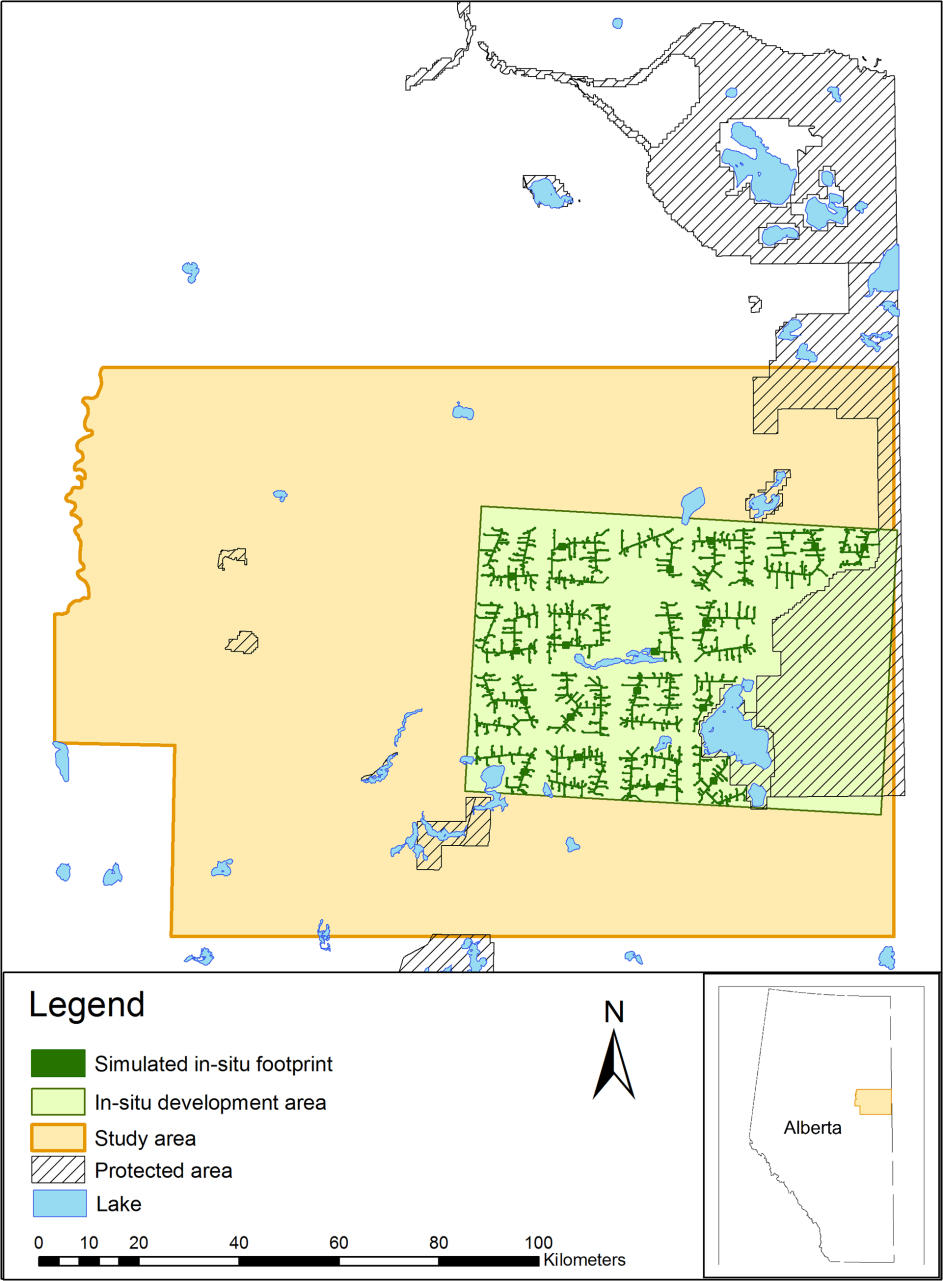
In-situ oil sands development scenarios simulated to test development effects on caribou movement, showing *in-situ* oil sands developments with a 2-km buffer between projects.

Previous studies of caribou behaviour have shown reduced movements caused by pipelines, but a complete barrier can be avoided by elevating pipelines [15, 16]. The emerging issue with ISDs is that the density of pipelines is expected to be many times greater than historic oil-transport pipelines because ISDs form an intensive network of above-ground pipelines that are used to extract bitumen from the ground (Fig 1). Consequently, maintaining unrestricted caribou movement has become an objective of the Governments of Canada and Alberta [8] with subsequent regulatory requirements that must be met by industry (i.e., providing crossing structures and pathways across above-ground pipelines). However, current densities of ISDs are low at the scale of caribou home ranges in boreal Alberta, making it difficult to gather data to evaluate whether ISDs actually block caribou movements. Thus, landscape-level simulations are needed to predict the future extent of ISDs and whether these will substantially alter caribou movements. Simulating a range of future development scenarios to predict ecological impacts is commonly used with forest management [17–19] but has rarely been applied to oil and gas development, despite the rapid increase of this activity.

Here we test for effects of current and simulated future (i.e., 50 years from now) ISDs on simulated caribou movements. We first developed an algorithm to simulate future ISDs in collaboration with industry managers who provided typical development plans. We varied the spacing between ISDs (no buffer, 800-m buffer and 2-km buffer), whether or not there was a large protected area in regional planning, and the permeability (impermeable to completely permeable) of ISDs, using 30 different scenarios. We then tested how much these treatments influenced caribou step lengths (the distance between two successive locations) and annual home range size (the space an animal occupies over the course of a year), key metrics of small and large spatiotemporal scales of movement, respectively [11, 20]. The effects of ISD spacing and permeability, and protected areas on caribou movement were tested because these were hypothesized to influence caribou movement and could realistically be managed by land-use managers and ISD developers.

As a means of validating the simulations, we then tested whether current above-ground pipelines are sufficiently permeable to maintain caribou step lengths and annual home ranges in the future. We compared simulated expected number of caribou crossings of above-ground pipelines to actual crossings measured by camera traps deployed in the study area to calculate the ratio of successful crossings. Caribou movements were parameterized with existing location data from Global Positioning System (GPS)-collared individuals in Alberta and an existing caribou habitat model using a step selection function (SSF) approach [21].

Our simulations provide land-use planners with the ability to prioritize the most efficient means of mitigating potential effects of ISDs on caribou movement by contrasting the benefits of ISD permeability and spacing, or the use of protected areas. Furthermore, our results indicate whether the current permeability of above-ground pipelines provides adequate crossing rates for caribou to maintain step lengths and home ranges in the future.

## Methods

### Study Area

Our study occurred in an 18,000-km^2^ area of northeast Alberta, Canada (Fig 1) that is currently being developed to extract bitumen from *in-situ* oil sand deposits. The current technology used to extract bitumen “*in-situ*” involves steam assisted gravity drainage (SAGD), where steam is pumped via wells belowground to heat bitumen, rendering it less viscous and thus more easily pumped to the surface. This requires up to five 34-cm to 50-cm diameter pipelines bundled together on support racks to transport steam to each well from a central processing facility, and bitumen from the well back to the facility [22, 23]. These above-ground pipelines and associated infrastructure are expected to remain on the landscape for at least 50 years. Vegetation within the study area consists of typical boreal forest species including black spruce (*Picea mariana*) in lowlands and aspen (*Populus tremuloides*) and mixed deciduous and coniferous forest in uplands, with an extensive network of bog, marsh and fens.

Densities of caribou predators and their alternate prey are likely moderate to high and increasing in the study area. Wolves (*Canis lupus*) were previously estimated in the region at 11.5/1,000 km^2^ [6]. White-tailed deer (*Odocoileus virginianus*) density has also recently been increasing [6].

### In-situ Development Simulations

ISDs were simulated within a 4,770 km^2^ subset of the study area where development is expected to be most intensive. This area is approximately five times the size of an average caribou home range in the region (mean = 967 km^2^, SE = 237 km^2^). We used the spatial distribution of actual planned footprint within four *in-situ* leases (i.e., single ISD areas) as a basis for modelling and simulating future ISDs, in leases where planned footprint was unknown. Details of the algorithm used to simulate future ISDs are provided in S1 File.

ISDs were simulated using 30 different scenarios (S2 File). We simulated scenarios with different lease spacing including: (1) within actual existing leases (no spacing), (2) within 15,675 ha rectangle leases (i.e., the average actual lease size) spaced 800-m apart, and; (3) within 15,675 ha rectangle leases spaced 2-km apart (Fig 1). Spacing between leases is theoretically possible due to more expensive subsurface horizontal drilling technologies. These scenarios were simulated with and without the Lower Athabasca Regional Plan (LARP) protected areas proposed for the study area (Fig 1), where no further development was permitted [24]. We simulated full ISDs of all leases in the study area that is expected to occur within 50 years without any reclamation, as was deemed likely by industry partners. Simulated ISDs were converted to rasters with a 10-m spatial resolution and attributed a value on the logit scale to model relative permeability, including: complete permeability = 1, high permeability = 0.6, high-moderate permeability = 0.1, moderate permeability = 0.01, moderate-low permeability = 0.0001, low permeability = 0.00001, and impermeable = 0. These values were used to obtain even coverage along the independent axis where permeability was used to predict effects on caribou movement.

All simulated ISD scenarios were mapped and then qualitatively validated by nine *in-situ* oil sands companies operating in the study area. They agreed that these were plausible representations of what the landscape could look like in the future. In addition, we compared the number of wells simulated within leases to the actual number of planned wells as a quantitative means of evaluating the ISD simulations.

### Caribou Movement Simulation

#### Caribou data

We obtained location data from 19 GPS-telemetry collared female caribou from the East Side of the Athabasca River (ESAR; six individuals) and Richardson (13 individuals) caribou populations in northeast Alberta, Canada (56° 58′, –111° 08′). Caribou were collared with a netgun by the Alberta Government under strict adherence to the Government of Alberta’s Animal Care Protocol No. 008 and approved by the Alberta Wildlife Animal Care Committee. GPS data were screened by removing all locations with a 2-dimensional fix and a horizontal dilution of precision (DOP) > 12 (M. Russell, Alberta Government, pers. comm.). Locations were collected year-round from 2008 to 2011 at two-hour intervals. We calculated turning angles and the step length between locations using the movement.pathmetrics tool in Geospatial Modelling Environment (GME; [25]).

#### Simulated future caribou movements

**Fig 2:**
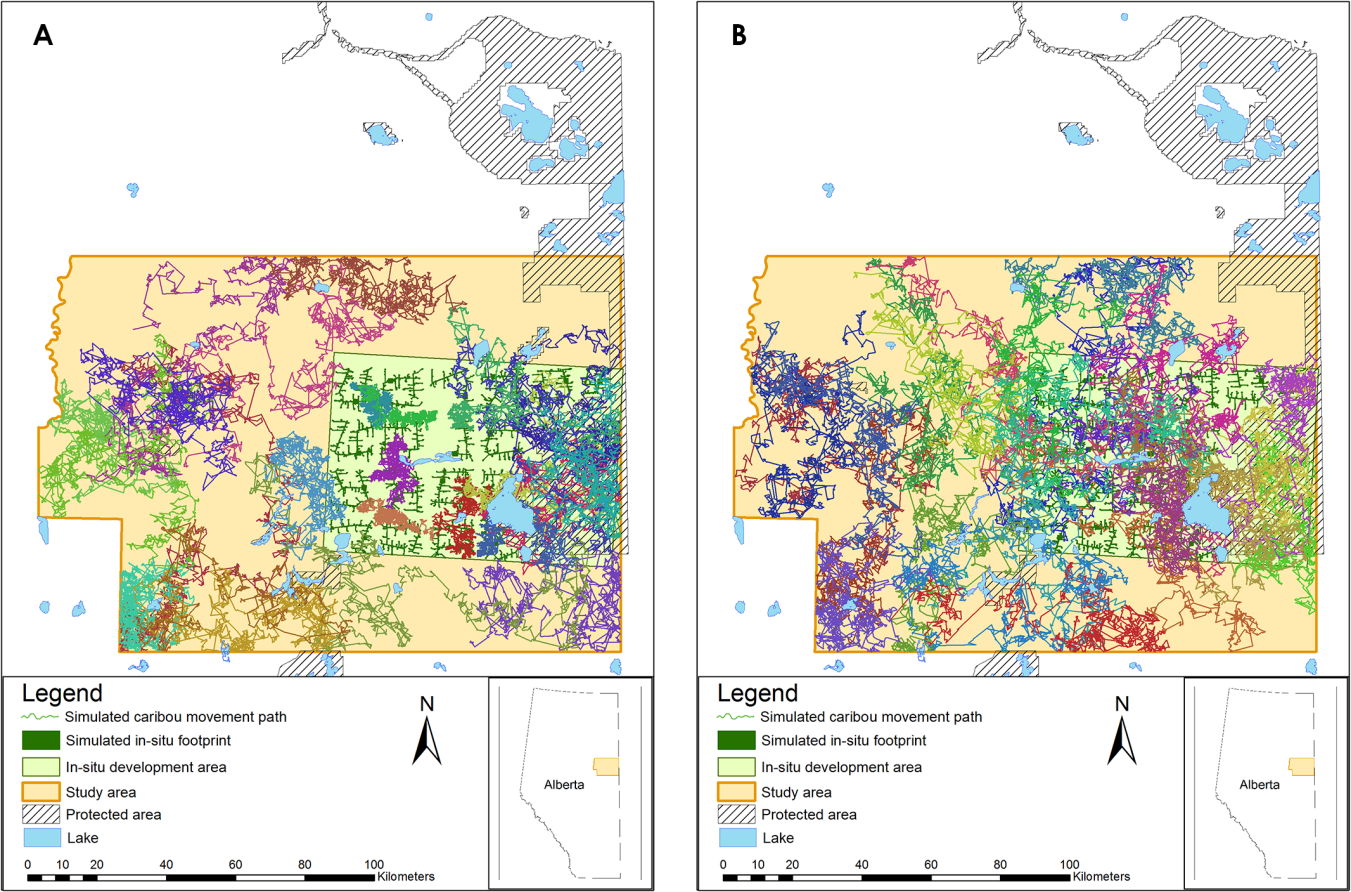
Simulated movements of 25 caribou over a one-year period relative to *in-situ* oil sands development that is modelled as completely impermeable (left) and completely permeable (right).

We simulated movements of 25 caribou over one year (steps every two hours, *n* = 4,380) within the study area under the different ISD scenarios (S2 File) using the movement.ssfsim1 tool in GME [25]. We found that 25 individuals was an adequate sample because in an initial simulation of 100 individuals, an asymptote in standard deviation of home range size and step length was reached at approximately 12 individuals (S3 File). One random starting point per caribou was generated within the study area and the same start points were used in each scenario. Movement steps were simulated by drawing 100 random step lengths and turning angles from the distribution of actual caribou step lengths and turning angles from GPS-location data. Then the caribou movement model was applied to calculate the relative probability of selecting each simulated step based on the underlying values of a resource selection function (RSF) model calculated for boreal caribou in Alberta (S4 File). The RSF provided a robust, validated model of the relative value of habitat for caribou in the study area (S4 File). Habitat covariates in the RSF model included the normalized difference vegetation index (an index of plant productivity modelled with a quadratic covariate), wetland type (including fen, bog or other), landcover type (including water, shrub, grass, conifer forest, deciduous forest or other), if the area was burned in the last 40 years or not, distance to soft linear feature (e.g., a pipeline or seismic line), distance to industrial site (e.g., an oil and gas facility) and distance to forestry cutblock. In addition to the RSF input covariate, we included an ISD covariate in the SSF model. ISD covariate values were generated on the logit scale for each modelled permeability scenario (see above). Thus, in scenarios with less permeable ISDs those steps crossing ISDs were less likely to be selected than those in scenarios with more permeable ISDs (Fig 2). We measured the number of times each simulated caribou crossed ISDs for each scenario and calculated the average number of crossings per scenario as an indicator of ISD permeability. The simulated caribou movement locations for each scenario are available from DRYAD (**https://datadryad.org/**; doi:10.5061/dryad.tp736).

### Testing the Effects of In-situ Development Permeability and Lease Spacing, and Protected Areas on Future Caribou Movement

We calculated generalized linear mixed models (GLMMs) of simulated caribou home range and step length as a function of ISD permeability, *in-situ* lease spacing, and protected areas using a normal link function to test for and compare relative effects of each on caribou movement [26]. We calculated GLMMs with all combinations of covariates, including models with and without a squared term for number of crossings to test for a non-linear relationship. We used all combinations of covariates, as there were few variables and we treated each variable as a hypothesis [27]. We also included a random effect for start point, because where a simulated caribou started on the landscape could affect how many times it crossed a development. For example, if a caribou started in the center of the ISD area, it was more likely to interact with and potentially cross an ISD then a caribou that started outside of the ISD area. We ranked models using Akaike’s Information Criteria (AIC), where the model with the lowest AIC value and models with a difference in AIC value less than two from the lowest AIC model were considered the most parsimonious for predicting caribou home range and step length [28].

### Estimating the actual Permeability of Current Above-ground Pipelines to Caribou

We obtained data on actual caribou crossings of above-ground pipelines (AGPs) using camera traps. AGPs are a subset of ISDs but comprise the vast amount of their linear footprint and are therefore the focus of caribou crossing mitigation. Camera traps were deployed on AGPs at three separate ISDs between 26 September 2006 and 14 January 2013 by Golder Associates Ltd. Only one AGP was regularly crossed by caribou (i.e., had >4 caribou crossings throughout the entire monitoring period). Therefore only data from this project were used in this analysis. The two ISD projects that were not crossed by caribou were either in lower quality caribou habitat as estimated by the RSF, or were <5-km long so there was opportunity for caribou to go around the ISD.

Reconyx cameras with motion sensors and infrared flashes were deployed at each crossing site. Cameras were either placed on a large diameter tree at locations with AGP heights between 1.0-m and 2.5-m from the ground to monitor under-pipe crossings, or at crossing structures on an AGP support structure 1-m from the ground to monitor over-pipe crossings. Twenty-five cameras were placed at under-pipe crossings, and six at over-pipe crossings.

#### Current caribou movement simulation

Caribou movement was simulated during continuous camera trap sampling periods. Camera data were divided into periods of continuous sampling by camera traps, when groups of cameras (i.e., >10 cameras) were concurrently running continuously to ensure that no caribou crossing events were missed during sampling.

Caribou movement was simulated using the caribou movement model previously described. The total number of simulated steps per caribou was equivalent to the length of the sampling period. Movement was simulated within the extent of the Cold Lake Air Weapons Range (CLAWR) and ESAR Christina boreal caribou herd boundaries in Alberta. Simulations were conducted for two caribou population sizes, ‘low’ and ‘high.’ Low population size (160 caribou) was calculated as the current estimated number of caribou within the CLAWR (~150 caribou) and ESAR (~120) herds, adjusted for area of the Christina herd (8.6% of ESAR). There was concern that the caribou population in northeast Alberta may be underestimated [29], therefore we simulated a scenario with double the current population estimate (320 caribou) as a high population size. We randomly generated 160 and 320 starting locations within the study area for the low and high population size scenarios, respectively.

#### Actual Above-ground Pipeline crossings by caribou

After each current caribou movement simulation, we calculated the number of times a caribou movement path intersected with a camera trap ‘detection area.’ We simulated two different detection areas. One detection area was equivalent to the size of the detection area cone for Reconyx cameras, a 324.1-m^2^ area (a radius of 10-m around a camera) where a passing animal is detected by the camera motion sensor [30]. However, animals may have been attracted towards cameras from a larger area due to the topography or presence of crossing structures along AGPs designed to promote crossing at those locations. Any animal intending to cross the AGP may have been drawn from a larger area towards a camera location. Therefore, we also simulated a detection area with a 100-m radius around the camera trap and measured the number of simulated detected AGP crossings in that area. We did this to account for simulated crossings that may have occurred near the camera site but were not detected in the simulated detection cone because our caribou movement model did not account for the potential attraction towards a crossing structure.

The ratio of successful AGP crossings by caribou was calculated as the number of actual crossings detected by camera traps divided by the number of simulated crossings during the same period, where a value >1 indicates more crossings than expected and a value <1 indicates fewer crossings than expected.

## Results

### In-situ Development Simulation

Our ISD model accurately simulated the number of wells within developed ISD lease boundaries. The average number of wells simulated within a lease over 100 iterations (mean = 75, mode = 90–95) was similar to the number of actual wells (94) within leases.

### Resource Selection Function Model

The RSF model was validated using independently collected caribou VHF telemetry location data (S4 File). Validation correlation coefficients, which indicate if and how well the area-adjusted frequency of RSF scores measured at VHF locations were correlated with the rank of the RSF score were *r*_*s*_ = 0.70 and *r*_*s*_ = 0.82 for summer and winter RSF models, respectively. This indicates that independent caribou VHF locations more frequently occurred in higher-ranked RSF bins, suggesting the model predicted where caribou were more likely to occur.

### Effect of In-situ Development Permeability on Caribou Movement

**Fig 3:**
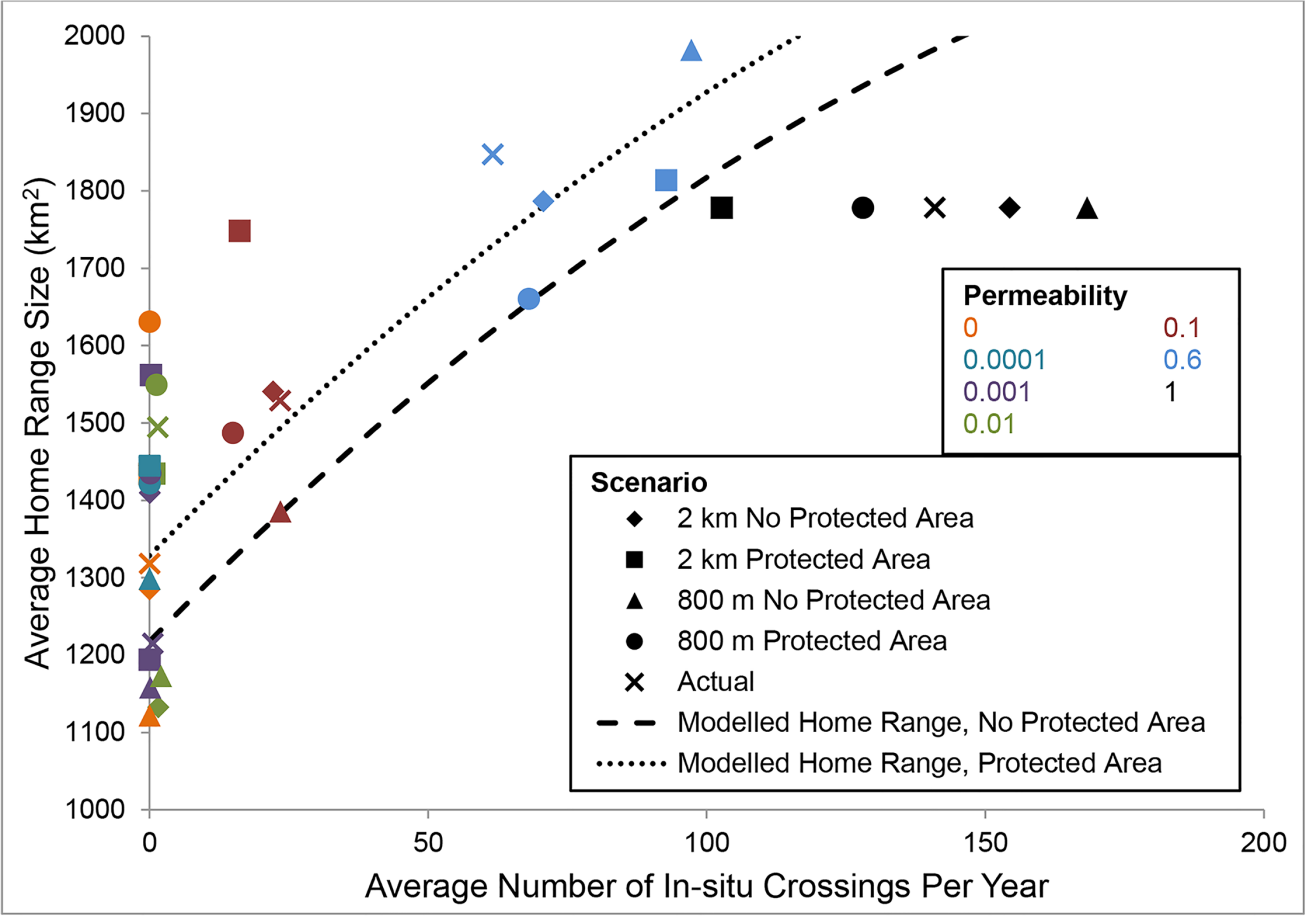
Average simulated caribou (n = 25) home range size as a function of the number of caribou crossings of *in-situ* developments (ISDs) in the study area under different spacing, protected area and modelled ISD permeability scenarios. Scenarios are indicated by different markers and permeability is indicated by colour. The predicted relationship between home range size and number of crossings, as determined using a generalized linear mixed model (Table 2), is also indicated for scenarios with (dotted line) and without (dashed line) protected areas.

**Fig 4:**
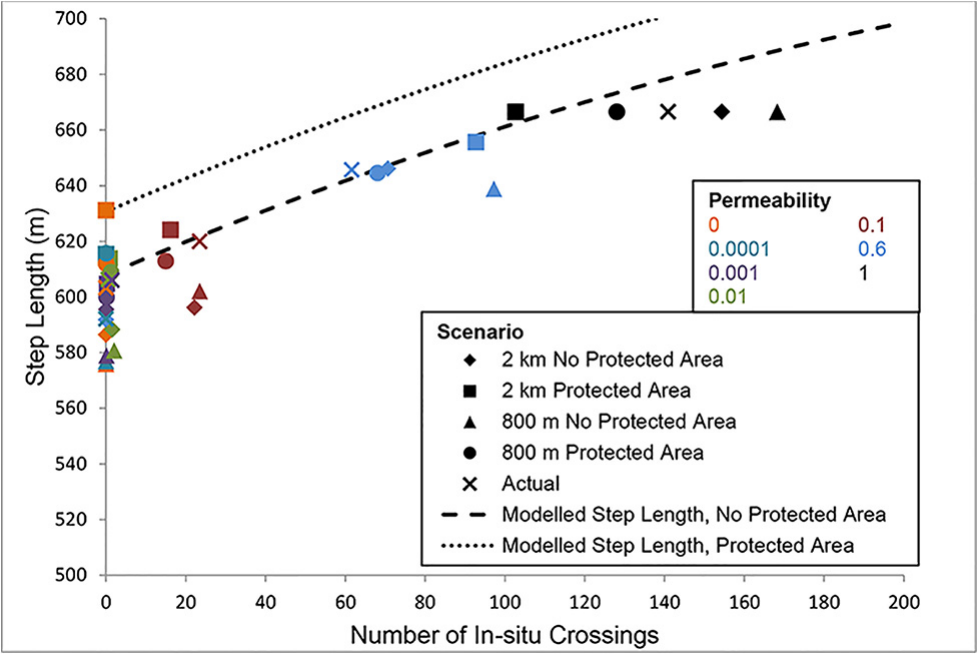
Average simulated caribou (n = 25) step length as a function of the number of caribou crossings of *in-situ* developments (ISDs) in the study area under different spacing, protected area and modelled ISD permeability scenarios. Scenarios are indicated by different markers and permeability is indicated by colour. The predicted relationship between home step length and number of crossings, as determined using a generalized linear mixed model (Table 2), is also indicated for scenarios with (dotted line) and without (dashed line) protected areas.

The permeability of simulated future ISDs had an effect on both caribou home range size and step length. In general, home range sizes and step lengths were significantly smaller and shorter in size and length, respectively, as ISD permeability decreased (Figs 3 and 4). Caribou home ranges and step lengths were each treated as dependent variables in separate analyses to determine how they were influenced by ISD permeability (measured as the number of successful ISD crossings by caribou), protected areas, and lease spacing. A squared term for number of crossings was included in the most parsimonious model for predicting caribou home range size, indicating that crossings resulted in a larger home range size, as its effect was roughly asymptotic for the range of data examined (Fig 3; Tables 1 and 2). Predicted home range size based on the GLMM decreased by 197 km^2^ (9%) as the number of crossings decreased from 150 to 100, 462 km^2^ (22%) as the number of crossings decreased from 150 to 50 and 796 km^2^ (37%) as the number of crossings decreased from 150 to 0 (Fig 3).

**Table 1:** Akaike Information Criteria (AIC) scores, differences and weights comparing models of caribou home range size and step length as a function of the number of *in-situ* development (ISD) crossings (permeability), protected areas and ISD lease spacing.

	Home Range			Step Length		
Model	AIC	ΔAIC	AIC weight	AIC	ΔAIC	AIC weight
Crossings + Crossings^2^ + Protected Area	13,863.9	0.0	0.622	9,341.8	16.5	0.000
Crossings + Crossings^2^ + Protected Area + Spacing	13,865.4	1.5	0.294	9,325.3	0.0	1.000
Crossings + Crossings^2^	13,868.2	4.3	0.072	9,366.4	41.1	0.000
Crossings + Crossings^2^ + Spacing	13,871.8	7.9	0.012	9,363.9	38.6	0.000
Crossings + Protected Area	13,938.9	75.0	0.000	9,377.5	52.2	0.000
Crossings + Protected Area + Spacing	13,941.6	77.7	0.000	9,364.4	39.1	0.000
Crossings	13,943.9	80.0	0.000	9,402.5	77.2	0.000
Crossings + Spacing	13,947.1	83.2	0.000	9,400.7	75.4	0.000
Protected Area	14,012.9	149.0	0.000	9,587.3	262.0	0.000
Protected Area + Spacing	14,016.2	152.3	0.000	9,581.2	255.9	0.000
Spacing	14,019.1	155.2	0.000	9,602.3	277.0	0.000

**Table 2:** Coefficients and standard errors for the most parsimonious generalized linear mixed models predicting caribou home range size and step length.

	Home Range		Step Length	
Covariate	β	SE	β	SE
Intercept	1217.703	126.426	607.900	11.690
Crossings	7.368	0.869	0.612	0.049
Crossings^2^	-0.014	0.002	-0.001	0.000
Protected Area	110.752	44.204	22.790	3.537
800-m Spacing	-29.903	28.278	-21.200	4.683
2-km Spacing	-21.334	24.115	-13.640	4.689

The non-linear relationship between permeability and home range size suggests that at about 20 to 60 crossings per year, home range size is maintained, but at fewer crossings, home range size drops off significantly. Under a completely permeable scenario, the mean number of crossings was 141. This pattern suggests that approximately 14% to 43% (20/141 to 60/141 crossings) of crossings (relative to an undisturbed landscape) are needed to maintain current home range sizes (Fig 3). The presence of protected areas had a relatively small effect on home range size (111 km^2^or 5% increase). Spacing was included in the second ranked GLMM (Table 1), but the model-averaged coefficient had a large standard error and a negative effect (Table 2), i.e., counter to our hypothesis of a positive effect, indicating the effect of spacing was not precisely or accurately modelled by the GLMM.

Predicted step length decreased by 21-m (3%) as the number of crossings decreased from 150 to 100, 45-m (6%) as the number of crossings decreased from 150 to 50 and 74-m (11%) as the number of crossings decreased from 150 to 0. Protected areas had a smaller effect than crossings on predicted step length, which increased by 23-m (3%) in scenarios with a protected area compared to scenarios without. Like with the home range model, although the effect of spacing was included in the top-ranked GLMM (Table 1), the effect was counter to our hypothesis (a negative effect) and had a large standard error relative to the effect size (Table 2) and therefore the effect of spacing was considered unreliable in the GLMM.

### Actual Caribou Movement and Above-ground Pipeline Crossing Rates

The number of actual caribou crossings of AGPs detected by camera traps ranged from 1 to 143, depending on the sampling period (Table 3). The majority (85%) of these crossings were under-pipe crossings (caribou crossed underneath the pipeline, typically at natural topographical depressions), although most (81%) cameras were placed at under-pipe crossing structures.

**Table 3:** The number of actual and simulated expected aboveground pipeline (AGP) crossings by caribou. Ratios of actual crossings (measured by camera traps) to expected crossings greater than one indicate higher AGP permeability than expected. Simulated crossing rates were estimated using a 10-m or 100-m detection radius around cameras.

								Simulated Number of Crossings				Ratio of Actual to Simulated Crossings			
								10-m radius		100-m radius		10-m radius		100-m radius	
Period	Start Date	End Date	Days	Cameras	Over-pipe Crossings	Under-pipe Crossings	Actual Crossings	160 caribou	320 caribou	160 caribou	320 caribou	160 caribou	320 caribou	160 caribou	320 caribou
1	18/02/2010	26/04/2010	67	20	0	17	17	2	6	38	76	8.50	2.83	0.45	0.22
2	18/02/2010	11/12/2011	661	10	23	120	143	52	91	463	943	2.75	1.57	0.31	0.15
3	10/02/2012	27/03/2012	46	13	0	1	1	1	8	20	65	1.00	0.13	0.05	0.02
4	10/02/2012	19/11/2012	283	11	0	9	9	27	51	250	250	0.33	0.18	0.04	0.04
											Mean	3.15	1.18	0.21	0.11

The number of actual caribou AGP crossings detected increased with the number of predicted crossings (Table 3). At the current (i.e., low) predicted caribou population size, and considering a 10-m detection radius for cameras, actual caribou AGP crossings were on average 3.15 times more frequent than expected, with a maximum 8.50 and minimum 0.33 ratio of actual to simulated expected crossings (Table 3). At twice the current estimated (i.e., high) caribou population size, actual AGP crossings were also greater than expected on average (a 1.18 ratio of actual to expected crossings). We found that six out of sixteen caribou crossing simulation scenarios (and four out of eight scenarios that used current caribou population estimates) were above a 0.43 ratio of actual to expected crossings (0.43 was the maximum level at which home range sizes declined relative to a completely permeable AGP scenario).

The number of simulated expected crossings was an order of magnitude larger when a 100-m radius camera detection area was considered compared to a 10-m detection radius (Table 3). Using a 100-m detection radius for cameras and the low population size, the ratio of actual to simulated expected crossings was on average 0.21, with a maximum of 0.45 and minimum of 0.04. At a 100-m radius and high population size, the ratio of actual to simulated expected crossings was on average 0.11, ranging from 0.02 to 0.22.

## Discussion

We examined how three factors relating to *in-situ* oil sands developments could affect future caribou movement: permeability across ISDs, spacing between ISDs, and the inclusion of protected areas in regional planning. Permeability across ISDs was the main factor affecting caribou home range size. Permeability had an approximately two to seven times larger effect on caribou home range size and step length than protected areas, and lease spacing had no effect. Furthermore, the effect of permeability on caribou home range size became stronger at low permeability, suggesting that a minimum level of permeability is needed to reduce effects on caribou home range size and step length. This non-linear relationship is important because it means that a modest improvement to permeability will provide a higher than proportional benefit to caribou movement.

The patterns we observed are intuitive when considered in the context of our simulated landscape. Regardless of whether 800-m or 2-km lease spacing is implemented, ISDs are likely to be prominent features on the landscape in 50 years. Therefore, permeability across ISDs will be the greatest factor dictating caribou space use. Protected areas will also have an effect on caribou movement, because they provide less developed areas that caribou can move through freely, but will have less of an effect than permeability, again because ISDs have the potential to encompass much more space than protected areas. The relative effect of protected areas will undoubtedly be roughly proportional to their size, so if protected areas dominate the landscape then they will have a greater influence on caribou movement, compared to the other factors we evaluated.

In practical terms, if caribou movement across the landscape is restricted by ISDs to *c*. less than 14% to 43% of unrestricted movements, caribou may be incapable of maintaining their home ranges and fine-scale movements. Human developments such as road building and forest harvesting have also been shown to affect caribou home range size with potentially negative consequences for caribou persistence [20]. If ISD permeability tends to 0 in the absence of mitigation, the decrease in home range size will be severe (37%). The implication of restricting movement includes reduced availability of resources, such as food, and more importantly for woodland caribou, finding predator-free space. For species such as caribou, which move long distances across a diverse matrix of habitat, habitat fragmentation may increase susceptibility to mortality [31]. Specifically, restricting movements may have implications for caribou encountering predators [32, 33]. Based on the GLMM models, results were less severe when evaluating effects on caribou step lengths. However, the effect of permeability on step length has similar implications relating to constraints on caribou movement.

Results of future ISDs and caribou movement scenarios are ideally considered within the context of current caribou movement and AGP crossing rates. We found that for half of caribou movement simulations based on current population estimates, the crossing ratio was greater than the 43% level necessary for caribou to maintain home ranges and step lengths in the future. This suggests that currently, AGP permeability may be at or close to the level needed for caribou to maintain their home ranges sizes and step lengths. Efforts should be placed on maintaining permeability across AGPs rather than potentially more costly methods of increasing lease spacing by using horizontal subsurface drilling technology.

Current caribou crossings of AGPs were rarely detected by cameras (approximately once every one-hundred to one-thousand camera days). Our movement simulations confirm that caribou can currently be expected to rarely cross AGPs. Notably, caribou appeared to use each crossing structure types based on their availability. Nevertheless, the current mitigation of maximizing AGP heights over natural depressions in the landscape appears to be an effective means to maintain landscape permeability for some projects, reducing the need for more expensive above-pipe crossing structures. If maintaining caribou movement is the sole objective for crossing structures, then under-pipe crossings may be an effective means to maintain permeability of AGPs.

There were limitations to our future scenario modelling, most notably that we did not model whether the effects of ISDs on caribou movement would ultimately affect caribou fitness or survival (e.g. [34]). Rather, we infer that restricting caribou movement will have negative consequences for caribou survival, as animal movement is linked to population dynamics [35, 36] via access to resources, including predator-free space. As well, it is clear that the ideal approach to predict ISD impacts would be based on empirical data, but this analysis is not possible because of the limited extent ISDs have on the landscape relative to caribou home range size and low caribou density [3]. Simulations are the only tool at our disposal, and are important especially because ISDs cannot be easily removed once they are in place.

For AGP crossing simulations, the effective detection area of cameras and caribou population size were both important considerations. Obviously, a larger detection radius and/or larger caribou population size resulted in a greater number of simulated AGP crossing events and thus a lower ratio of successful crossing. The ratio of successful crossings was generally proportional to population size, and in the 10-m camera detection radius scenario, a larger population did not result in an average crossing ratio <1.00, suggesting that even if the caribou population is twice as large as currently estimated, caribou movement may not be significantly restricted by AGPs. However, when the effective detection radius was 100-m, the proportion of successful crossings went below the 0.43 level for all but one scenario, regardless of population size. The effective detection radius of cameras may therefore be a key parameter to using cameras to measure crossing rates of AGPs. Unfortunately, data are not available to determine the effective detection radius of cameras for this study. Therefore, it is suggested that as caribou or other large mammals increasingly interact with ISDs, predictions of future permeability presented with these simulations continue to be validated periodically using camera and GPS radio-collar data from caribou and other wide-ranging species.

## Supporting Information

S1 File Methods used to simulate the occurrence of future *in-situ* developments. (DOCX)

S2 File *In-situ* development simulation scenarios. (DOCX)

S3 File Relationship between the number of simulated individual caribou home ranges and step lengths and the standard deviation of average home range and step length sizes. (DOCX)

S4 File Resource selection function model used to simulate caribou movement. (DOCX)
