# Supplementary material for: Influence of In-Situ Oil Sands Development on Caribou (Rangifer tarandus) Movement
Source: PLoS One. 2015 Sep 8;10(9):e0136933. doi: 10.1371/journal.pone.0136933 (PMC4562618; doi:10.1371/journal.pone.0136933)
Supplement: S1 File — (DOCX) [file pone.0136933.s001.docx]

# Appendix S1. Methods used to simulate the occurrence of future in-situ developments.

Using the lease boundary and well pad locations from existing ISDs, a point pattern object (NLppp) was created for analysis in the R (R Development Core Team 2012) package ‘spatstat’ (Baddeley et al. 2010). A spatial logistic regression model (slrm) that used a discretized pixel grid with 1 assigned to pixels with a well and 0 assigned to pixels with no well was fitted to the NLppp. We then used the fitted model to simulate well distributions within lease boundaries where footprint was unknown. A 244-m × 191-m rectangle (i.e., the average well pad size within the known proposed ISDs) was created around each simulated point centroid to represent the simulated well pad footprint.

A linear feature network (roads and AGPs) connecting all simulated well pads within each simulated lease was generated in a series of steps. First, the well distribution was separated into three clusters using the ‘partitioning –around-medoids’ function in the R package ‘cluster’ (Maechler et al. 2012) and an ellipse enclosing one standard deviation was drawn around each cluster. We intersected the ellipse with a line to generate a trunk line through each cluster. To connect trunk lines to each other and to simulated well pads, a Cost Distance/Cost Path was calculated with a fishnet cost raster in which cross hatched ‘on-grid’ lines were slightly less expensive than the enclosed ‘off-grid’ squares (ratio 10/12). The line raster was buffered by 62 m (the average width of linear features in the actual proposed ISD data). Central processing facilities were simulated by creating a 1.2-km square feature (i.e., their average size) at the midpoint of the first trunk line.

**References**

Baddeley, A., M. Berman, N. Fisher, A. Hardegen, R. Milne, D. Schuhmacher, R. Shah, and R. Turner. (2010) Spatial logistic regression and change-of-support in Poisson point processes. *Electronic Journal of Statistics*, 4, 1151–1201.

Maechler, M., P. Rousseeuw, A. Struyf, M. Hubert, K. Hornik. 2012. Cluster: cluster analysis basics and extensions. R package, version 1.14.3.
