# Supplementary material for: Influence of In-Situ Oil Sands Development on Caribou (Rangifer tarandus) Movement
Source: PLoS One. 2015 Sep 8;10(9):e0136933. doi: 10.1371/journal.pone.0136933 (PMC4562618; doi:10.1371/journal.pone.0136933)
Supplement: S2 File — (DOCX) [file pone.0136933.s002.docx]

# Appendix S2. In-situ development simulation scenarios.

Table A1. Simulation scenarios used to measure the influence of *in-situ* development permeability and spacing, and protected areas on caribou home range size and step lengths.

| Scenario | | |
| --- | --- | --- |
| Permeability | **Lease Spacing (m)** | **Protected Areas** |
| 1 | N/A | N/A |
|  |  |  |
| 0 | 800 | No |
| 0.0001 | 800 | No |
| 0.001 | 800 | No |
| 0.01 | 800 | No |
| 0.1 | 800 | No |
| 0.6 | 800 | No |
|  |  |  |
| 0 | 800 | Yes |
| 0.0001 | 800 | Yes |
| 0.001 | 800 | Yes |
| 0.01 | 800 | Yes |
| 0.1 | 800 | Yes |
| 0.6 | 800 | Yes |
|  |  |  |
| 0 | 2,000 | No |
| 0.0001 | 2,000 | No |
| 0.001 | 2,000 | No |
| 0.01 | 2,000 | No |
| 0.1 | 2,000 | No |
| 0.6 | 2,000 | No |
|  |  |  |
| 0 | 2,000 | Yes |
| 0.0001 | 2,000 | Yes |
| 0.001 | 2,000 | Yes |
| 0.01 | 2,000 | Yes |
| 0.1 | 2,000 | Yes |
| 0.6 | 2,000 | Yes |
