# Supplementary material for: Influence of In-Situ Oil Sands Development on Caribou (Rangifer tarandus) Movement
Source: PLoS One. 2015 Sep 8;10(9):e0136933. doi: 10.1371/journal.pone.0136933 (PMC4562618; doi:10.1371/journal.pone.0136933)
Supplement: S3 File — (DOCX) [file pone.0136933.s003.docx]

**Appendix S3. Relationship between the number of simulated individual caribou home ranges and step lengths and the standard deviation of average home range and step length sizes.**

.
