# Supplementary material for: Influence of In-Situ Oil Sands Development on Caribou (Rangifer tarandus) Movement
Source: PLoS One. 2015 Sep 8;10(9):e0136933. doi: 10.1371/journal.pone.0136933 (PMC4562618; doi:10.1371/journal.pone.0136933)
Supplement: S4 File — (DOCX) [file pone.0136933.s004.docx]

**Appendix S4. Resource selection function model used to simulate caribou movement.**

**Modelling Resource Selection of Boreal Caribou in Alberta**

By Craig DeMars, Robert Serrouya, and Tyler Muhly

**Methods: Second-Order Selection (individual home ranges compared to herd ranges)**

**Caribou Spatial Data**

We used GPS location data from 152 adult female caribou distributed among eight independent herds: East Side of the Athabasca River (ESAR), West Side of the Athabasca River (WSAR), Cold Lake, Slave Lake, Nipisi, Richardson, Red Earth and Chinchaga. These data were collected by Alberta Environment and Sustainable Resource Development (AESRD), Government of Alberta, during their caribou monitoring program from 1998 to 2012. Prior to analyses, the data were screened to remove all locations with low positional accuracy (e.g. < three-dimensional fixes and a horizontal dilution of precision > 12, M. Russell, Government of Alberta, pers. comm.; Lewis *et al.* 2007). We also used VHF location data collected during 1991-2012 from 606 individual adult female distributed among all northern Alberta boreal caribou herds. Positional accuracy of the VHF data was estimated to be < 250-m and this data set totalled 4,098 locations.

**Sampling Framework**

We evaluated for seasonal differences in resource selection by first partitioning the data into two seasons: summer, from May 15th to September 30th and winter, from October 1 to May 14. The summer data consisted of 280,970 GPS locations from 123 individuals with the number of locations per individual varying from 27 to 10,394. The winter data set had 327,070 GPS locations from 150 individuals with the number of locations per individual varying from 50 to 12,212. To assess how female caribou selected resources within a herd’s range (i.e. second-order selection, *sensu* Johnson 1980), we compared caribou GPS locations (i.e. “used” locations) to randomly generated locations within herd ranges (i.e. “available” locations). For each GPS location, we generated one random location within the range of the individual's herd.

**Resource Variables**

We modelled resource selection using explanatory variables representing vegetation characteristics (cover type and normalized difference vegetation index [NDVI]), fire history and anthropogenic footprint. NDVI is a measure of green vegetation in the landscape and has been used as an index of forage quantity and/or quality for ungulates. For land cover, we used remotely sensed vegetation cover data from the Alberta Biodiversity Monitoring Institute (ABMI Remote Sensing Group 2012). This data indicates dominant vegetation cover type (categories: conifer forest (lc_confor), broadleaf forest (lc_decfor), mixed forest(reference category), shrubland (lc_shrub), grassland (lc_grass), water, and other (lc_other2), which included agriculture, exposed land, rock/rubble, developed and snow/ice) derived from Landsat data collected in year 2000 at 0.5 ha to 2.0 ha spatial resolution. To specifically model wetlands, an important land cover for caribou, we used Alberta peatland inventory data (Halsey et al. 2003). This data contains information about wetland class, vegetation and landform mapped as polygons from aerial photos. For our analysis, we identified polygons with >50% bog as bogs (bog) and >50% fen as fens (fen). NDVI was measured from MODIS satellites at 16 day intervals at a 250 m pixel resolution (https://lpdaac.usgs.gov/products/modis_products_table/myd13q1). Average NDVI values were calculated at each pixel for each season (ndvi_act). We modelled fire history using mapped fire inventory polygons (Alberta Sustainable Resource Development 2012), which we further classified into fires ≤40 years old (recent burns) and fires >40 years old (fire40). To characterize anthropogenic footprint, we measured distance of each location to five different human footprint types: industrial (i.e., polygonal industrial sites; d_ind_act), hard linear (i.e., roads), soft linear (i.e., seismic and pipelines; d_sft_act), agriculture (i.e., cultivated and pasture lands) and forestry cutblock (d_cut_act). Footprint was obtained from the ABMI, which mapped the location of all footprint across Alberta in 2007 and 2010 using data from a variety of sources. Caribou locations from 2008 and earlier were attributed to 2007 human footprint data while caribou locations from 2009 and later were attributed 2010 human footprint data.

**Data Analysis**

We evaluated caribou resource selection by developing resource selection functions (RSFs; Manly *et al.* 2002). Prior to RSF development, we assessed for collinearity among resource variables by calculating Pearson correlation coefficients. For those variables that were highly correlated (*r* > 0.6) we removed the covariate least correlated with used locations. We also standardized all continuous variables prior to model fitting to facilitate comparison of relative effect sizes among resource variables.

We estimated RSFs using generalized linear mixed effect models (GLMMs; Zuur *et al.* 2009), which account for the hierarchical structure inherent in GPS location data. In all GLMMs we assigned individual caribou as a random grouping effect, which creates a random intercept for each caribou. We further developed two model structures that accounted for functional responses in selection (i.e., differential selection as a function of resource availability; Mysterud & Ims 1998). For the first, we specified random slopes for those variables identified as having a functional response (see below). This model structure has the form

g(x) = β_0_ + βx_ij_ + ... + β_n_x_nij_ + γ_nj_x_nj_ + γ_j_ (Gillies *et al.* 2006)

where γ_j_  is the random intercept for caribou *j* and γ_nj_ is the random slope (or coefficient) for caribou *j* with respect to covariate *x*_n_. The fixed-effects, or marginal, coefficients (βx_ij_) yield population-level inferences that can be interpreted within the classic use-availability design of

ω(x_i_) = exp(β_1_x_1_ +β_2_x_2_ + ...β_n_x_n_) (Manly et al. 2002)

where ω(x_i_) is the relative selection value of a sample unit (or pixel) in category *i* as a function of the explanatory covariates. For the second model structure, we developed generalized functional response models (GFRs; Matthiopoulos *et al.* 2011). GFRs account for functional responses in selection by including additional covariates describing resource availability for those resources identified as having a functional response. We assessed the performance of GFR variables in GLMMs that included random-intercepts only as well as in models that specified random slopes. Thus, in total we evaluated four RSF models for each season:

1. A random-intercept only GLMM without GFR variables
2. A random-intercept only GLMM with GFR variables
3. A random-intercept and random slope GLMM without GFR variables
4. A random-intercept and random slope GLMM with GFR variables

To identify covariates to include as functional responses, we first estimated individual RSFs by calculating logistic regression models with all covariates for each individual caribou included in the model. We then plotted the relationship between the availability of each covariate and the selection coefficient to determine if there was a functional response. A functional response was identified as a relationship that could be defined by a linear or quadratic equation with an R^2^ value > 0.1 and crossed zero (i.e., went from selection to no selection or vice-versa).

**RSF Model Performance and Validation**

We evaluated model fit and parsimony of the four seasonal RSF models using Bayesian Information Criterion (BIC) scores. We validated each RSF model with the independently collected caribou VHF locations following a method similar to *k*-fold cross-validation (Boyce *et al.* 2002). Specifically, we measured each RSF model score at each VHF location then binned them into ten bins with more-or-less equal sample sizes. We then divided the number of locations by the total area of that bin in the RSF map (i.e., the area-adjusted frequency). Finally, we calculated a Spearman correlation coefficient (*r*_S_) between the area-adjusted frequency and bin rank. Models with higher predictive power will have larger correlation coefficients. We identified the best seasonal model for describing caribou resource selection by comparing by model fit and parsimony (BIC scores) with predictive performance (*r*_S_ score).

**Results**

The RSF model indicated that caribou selected vegetation with intermediate NDVI values in summer, but selected higher NDVI values in winter. Caribou selected fen and bog wetland types over other wetland types, selected conifer forest (lc_confor) and shrub (lc_shrub) landcover types, and avoided deciduous forest (lc_decfor), water (lc_water) and other (lc_other2) landcover types relative to mixed forest landcover types in the in summer and winter. Caribou also selected grassland (lc_grass) cover types in the winter, but not in the summer. In both seasons, caribou avoided burns less than 40 years old (fire40). Caribou selected soft linear features (d_sft_act, e.g., seismic lines and pipelines) and avoided forestry cutblocks (d_cut_act) in the summer and winter. Caribou avoided industrial features (d_ind_act) in the summer but selected them in the winter.

Results of the RSF model are consistent with other second-order (i.e., home range) boreal caribou habitat selection studies. Boreal caribou typically avoid habitats expected to have higher predation risk, i.e., higher vegetation food quality and quantity habitats that support more ungulates and thus predators. Therefore caribou typically select home ranges consisting of low productivity, older conifer forest and peatlands (Bradshaw et al. 1995; Rettie and Messier 2000; McLoughlin et al. 2005; Courbin et al. 2009; Moreau et al. 2012), which is consistent with our findings. Consequently, caribou typically avoid forestry activity that creates early seral forests (Smith et al. 2000; DeCesare et al. 2012), which is also consistent with our findings. The selection of soft linear features that we found is counter to the theory that caribou may avoid linear features to mitigate predation risk. However, other studies of woodland caribou have found that avoidance of linear features may occur at smaller scales (i.e., within home range) then our study (DeCesare et al. 2012).

For the summer season, the predictive performance was highest for the full model, random intercept, but with no GFR (*r*_S_ = 0.70). This model had a predictive performance that was 9 units higher than the next best model (i.e. the random slope model that included a GFR). In winter however, 3 models had similar predictive performance scores: the random intercept full model with no GFR (*r*_S_ = 0.82), the full model with the GFR (*r*_S_ = 0.82), and random slope full model with the GFR (*r*_S_ = 0.83). For parsimony and for consistency we chose to use the same model structure as the summer model: i.e. the full model with random intercept and no GFR. Model details including parameter estimates are provided below.

Summer model: Yellow indicates the model that was used for the RSF mapping

Winter model: Yellow indicates the model that was used for the RSF mapping

**Literature Cited**

Boyce, M.S., Vernier, P.R., Nielsen, S.E. & Schmiegelow, F.K. (2002). Evaluating resource selection functions. *Ecological Modelling*, **157**, 281–300.

Bradshaw, C. J., Boutin, S., Hebert, D. M., & Rippin, A. B. (1995). Winter peatland habitat selection by woodland caribou in northeastern Alberta. Canadian Journal of Zoology, 73(8), 1567-1574.

Courbin, N., Fortin, D., Dussault, C., & Courtois, R. (2009). Landscape management for woodland caribou: the protection of forest blocks influences wolf-caribou co-occurrence. Landscape ecology, 24(10), 1375-1388.

DeCesare, N. J., Hebblewhite, M., Schmiegelow, F., Hervieux, D., McDermid, G. J., Neufeld, L., ... & Musiani, M. (2012). Transcending scale dependence in identifying habitat with resource selection functions. Ecological Applications, 22(4), 1068-1083.

Gillies, C.S., Hebblewhite, M., Nielsen, S.E., Krawchuk, M.A., Aldridge, C.L., Frair, J.L., Saher, D.J., Stevens, C.E. & Jerde, C.L. (2006). Application of random effects to the study of resource selection by animals. *Journal of Animal Ecology*, **75**, 887–898.

Halsey, L.A., D. Beilman, D.H. Vitt, S. Crow, S. Mehelcic and R. Wells. (2013). Alberta wetland inventory classification system 2.0. Alberta Sustainable Resource Development, Edmonton, AB, Canada, Pub. No. T/031.

Johnson, D.H. (1980). The comparison of usage and availability measurements for evaluating resource preference. *Ecology*, **61**, 65–71.

Lewis, J.S., Rachlow, J.L., Garton, E.O. & Vierling, L.A. (2007). Effects of habitat on GPS collar performance: using data screening to reduce location error: GPS collar performance. *Journal of Applied Ecology*, **44**, 663–671.

Manly, B.F.J., McDonald, L., Thomas, D.L., McDonald, T.L. & Erickson, W.P. (2002). *Resource selection by animals: statistical design and analysis for field studies*, Second edition. Kluwere Academic Publishers.

Matthiopoulos, J., Hebblewhite, M., Aarts, G. & Fieberg, J. (2011). Generalized functional responses for species distributions. *Ecology*, **92**, 583–589.

McLOUGHLIN, P. D., DUNFORD, J. S. and BOUTIN, S. (2005), Relating predation mortality to broad-scale habitat selection. Journal of Animal Ecology, 74: 701–707

Moreau, G., Fortin, D., Couturier, S. and Duchesne, T. (2012), Multi-level functional responses for wildlife conservation: the case of threatened caribou in managed boreal forests. Journal of Applied Ecology, 49: 611–620.

Mysterud, A. & Ims, R.A. (1998). Functional responses in habitat use: availability influences relative use in trade-off situations. *Ecology*, **79**, 1435–1441.

Rettie, W. J., & Messier, F. (2000). Hierarchical habitat selection by woodland caribou: its relationship to limiting factors. Ecography, 23(4), 466-478.

Zuur, A.F., Ieno, E., Walker, N., Saveliev, A.A. & Smith, G.M. (2009). *Mixed effects models and extensions in ecology with R*. Springer, New York, NY.
